# Dynamic posterior stabilization of shoulder hemiarthroplasty in long-standing neglected posterior dislocation of the glenohumeral joint

**DOI:** 10.4103/0973-6042.44145

**Published:** 2008

**Authors:** A. J. Shyam Kumar, Jeremy Oakley, Jamie Wootton

**Affiliations:** Orthopedic Specialist Registrar, Wrexham Maelor Hospital, Croesnewydd Road, Wrexham, LL13 7TD, UK

**Keywords:** Chronic, hemiarthroplasty, posterior shoulder dislocation

## Abstract

Posterior dislocations of the shoulder are rare. They account for less than 3% of all shoulder dislocations. The treatment of neglected bilateral posterior dislocation of the shoulder is controversial. We present a novel operative technique to stabilize a shoulder hemiarthroplasty that we used in the treatment of a chronic posterior dislocation of a shoulder with an acute four-part fracture of the proximal humerus.

## INTRODUCTION

Posterior dislocations of the shoulder are rare. They account for less than 3% of all shoulder dislocations.[1–3] The treatment of neglected bilateral posterior dislocation of the shoulder is controversial. Proposed treatment methods include shoulder hemiarthroplasty or acute osteochondral autografting.[4,5]

## CASE REPORT

**Figure 1:**
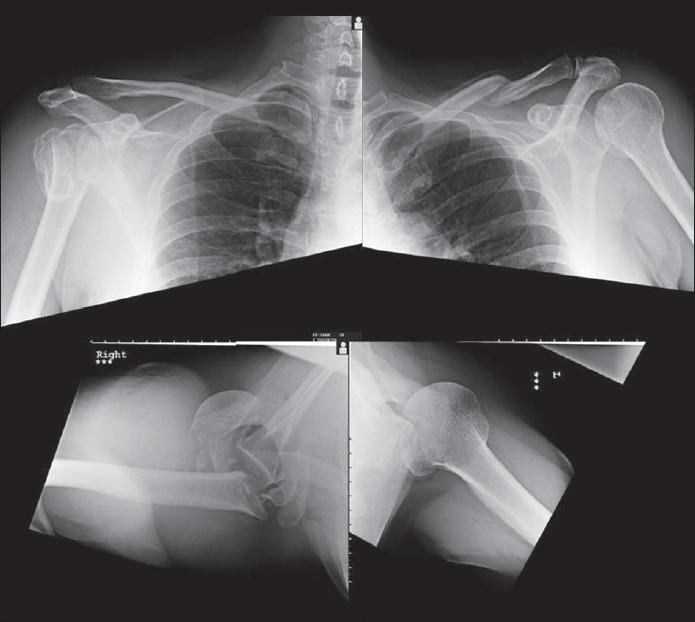
Preoperative radiograph of both shoulders

A 60-year-old male with mild learning difficulties presented to the clinic with increasing pain and difficulty in moving both shoulders for approximately 1 week. He denied any history of trauma. The patient was a known epileptic suffering from recurrent fits. Clinical examination revealed significant wasting of the deltoid bilaterally, with minimal bruising around both shoulders. There was limitation of movement of both shoulders in all directions due to pain. Distal neurovascularity was normal. Radiographs revealed chronically neglected bilateral posterior shoulder dislocations with an acute four-part fracture of the proximal humerus on the right side [Figure 1]. The patient and his family consented for surgery after a lengthy discussion. The right side was treated with a hemiarthroplasty with posterior stabilization using the long head of the biceps tendon (see technique below). Six weeks later, the left side was treated with open reduction and McLoughlin procedure.

## OPERATIVE TECHNIQUE

**Figure 2:**
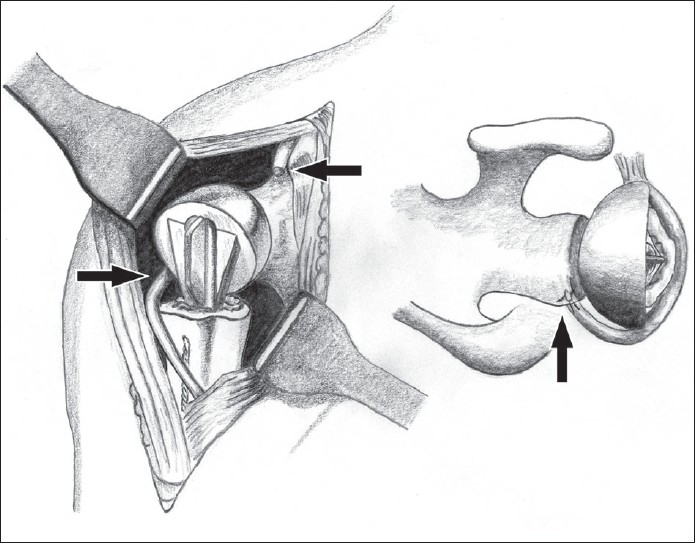
Illustration showing the transposition of the biceps tendon

**Figure 3:**
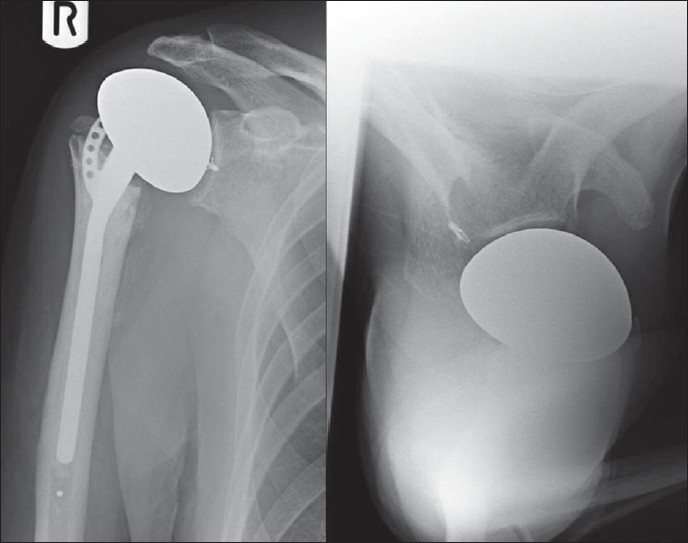
Postoperative radiographs of the right shoulder showing the prosthesis and the DePuy Mitek™ suture anchors *in situ*

In the beach-chair position access to the proximal humerus was obtained through the deltopectoral approach. The humeral head was found to be highly comminuted. The posterior capsule was virtually nonexistent, with the presence of a large cavity posterior to the glenoid indicating the chronicity of the posterior dislocation of the humeral head. The rotator cuff, along with its insertion into the greater tuberosity, was intact although moderately degenerate. A hemiarthroplasty was performed using the Equinoxe fracture stem (Exatech Ltd.) with the prosthesis inserted in 5° of retroversion. The greater and lesser tuberosities were reattached to the fracture stem using 5-0 ethibond sutures (Ethicon Ltd.). Despite the adequate retroversion, the prosthesis was grossly unstable posteriorly due to the nonexistent posterior capsule. The long head of the biceps was tenotomized close to its origin on the supraglenoid tubercle and was rerouted posteriorly. It was attached to the middle of the posterior rim of the glenoid using DePuy Mitek™ suture anchors (Johnson and Johnson Ltd.), thus creating a dynamic posterior restraint and preventing posterior dislocation of the prosthesis [Figures 2 and 3]. Postoperatively, the arm was rested in a polysling in internal rotation, with the forearm resting on the abdomen, for 2 weeks. Passive range of motion exercises were commenced at 2 weeks post-op, progressing to active assisted mobilization after a further 2 weeks. At 6 months' follow-up the patient was pain free and the shoulder remained stable despite a further episode of convulsions. His active forward elevation and abduction were 70° and 60°, respectively. Active external rotation was 20° and internal rotation allowed his hand to reach his upper lumbar spine. Functionally, he was able to reach his mouth for feeding himself and could brush his hair.

## DISCUSSION

As far as we are aware, this technique for posterior shoulder stabilization has not been previously reported in such a clinical situation. A similar technique has been described by Boyd and Sisk for recurrent posterior dislocation of the native joint but not for arthroplasty of the shoulder.[6] Posterior capsulorrhaphy has also been described for a posteriorly unstable shoulder arthroplasty.[7] This technique, however, cannot be used in situations where the posterior capsule is nonexistent due to the chronicity of the dislocation.
